# Resveratrol protects mice against SEB‐induced acute lung injury and mortality by miR‐193a modulation that targets TGF‐β signalling

**DOI:** 10.1111/jcmm.13542

**Published:** 2018-03-07

**Authors:** Hasan Alghetaa, Amira Mohammed, Muthanna Sultan, Philip Busbee, Angela Murphy, Saurabh Chatterjee, Mitzi Nagarkatti, Prakash Nagarkatti

**Affiliations:** ^1^ Department of Pathology, Microbiology and Immunology School of Medicine University of South Carolina Columbia SC USA; ^2^ Department of Environmental Health Sciences Arnold School of Public Health University of South Carolina Columbia SC USA

**Keywords:** 3,4,5‐trihydroxy‐trans‐stilbene, acute lung injury, death receptor 6, microRNA (miRNA/miR), miR‐193, pulmonary disease, resveratrol, staphylococcal enterotoxin B, transforming growth factor‐beta

## Abstract

Staphylococcal enterotoxin B (SEB) is a potent superantigen produced by *Staphylococcus aureus* that triggers a strong immune response, characterized by cytokine storm, multi‐organ failure, and often death. When inhaled, SEB can cause acute lung injury (ALI) and respiratory failure. In this study, we investigated the effect of resveratrol (RES), a phytoallexin, on SEB‐driven ALI and mortality in mice. We used a dual‐exposure model of SEB in C3H/HeJ mice, which caused 100% mortality within the first 5 days of exposure, and treatment with RES resulted in 100% survival of these mice up to 10 days post‐SEB exposure. RES reduced the inflammatory cytokines in the serum and lungs, as well as T cell infiltration into the lungs caused by SEB. Treatment with RES also caused increased production of transforming growth factor‐beta (TGF‐β) in the blood and lungs. RES altered the miRNA profile in the immune cells isolated from the lungs. Of these, miR‐193a was strongly induced by SEB and was down‐regulated by RES treatment. Furthermore, transfection studies and pathway analyses revealed that miR‐193a targeted several molecules involved in TGF‐β signalling (TGFβ2, TGFβR3) and activation of apoptotic pathways death receptor‐6 (DR6). Together, our studies suggest that RES can effectively neutralize SEB‐mediated lung injury and mortality through potential regulation of miRNA that promote anti‐inflammatory activities.

## 1 INTRODUCTION

Acute lung injury (ALI) is an inflammatory respiratory disorder characterized by injury to vascular endothelium and alveolar epithelium from the presence of pulmonary infiltrates which leads to hypoxia.^1^ Many different factors can lead to the development of ALI such as pneumonia, sepsis, age, trauma, smoke, exposure to toxic inhalants like humidifier disinfectants,^2^, ^3^ infectious bacteria such as *Pseudomonas aerogenosa*,^4^ opportunistic fungi like *Candida albicans* 5 and inhalation of bacterial toxins like staphylococcal enterotoxin B (SEB).^6^ During the initial infection or injury involving ALI, there is loss of alveolar‐capillary membrane integrity resulting in trans‐endothelial infiltration of immune cells, which causes the release of several cytotoxic and pro‐inflammatory mediators, such as interferon‐γ (IFN‐γ), interleukin‐6 (IL‐6), IL‐8 and tumour necrosis factor‐alpha (TNF‐α).^3^, ^7^, ^8^ The incidence and mortality rate of ALI remain high in the United States, as evidenced by the fact that over 200 000 patients developed the condition annually with 40% of these cases being fatal.^9^ SEB is a potent virulence factor produced by *Staphylococcus aureus* (*S. aureus*), a Gram‐positive bacterium.

Among the enterotoxins produced by *S. aureus*, SEB is the most well‐characterized and researched. Ingested SEB often leads to classic food poisoning symptoms in humans, while respiratory inhalation of SEB has the potential to be life‐threatening as it can induce toxic shock syndrome.^10^ Due to this, and the fact that SEB is extremely stable and easily aerosolized, the Centers for Disease Control (CDC) has listed this enterotoxin as a select agent, with potential to be used as a biological warfare agent.^11^, ^12^ The lethal nature of SEB is due to the fact that this toxin is a highly potent superantigen, classified as such because of its ability to activate a large proportion of T cells and thereby trigger massive amounts of pro‐inflammatory cytokines.^13^ SEB, like other superantigens, bypasses normal antigen processing and presentation by binding directly to the major histocompatibility complex‐II (MHC‐II) of antigen‐presenting cells (APCs). Then, the MHC‐II/SEB complex binds nonspecifically to T cells through the T‐cell receptor (TCR) and results in activation and proliferation of a large number of T cells.^14^, ^15^ Inhalation of this virulent factor into the lungs leads to massive activation of infiltrating immune cells, the release of several cytokines (termed a “cytokine storm”), destruction of the lung‐barrier architecture allowing increased tissue permeability and ultimately leading to the failure of normal respiratory function.^13^, ^16^, ^17^ Current treatment options for this condition are limited, often consisting only of supportive care or the use of immunosuppressive drugs with various negative side effects.^1^, ^18^ Therefore, it is essential that better treatment options are explored for ALI patients.

Resveratrol is a natural polyphenolic phytochemical product found in a wide variety of plants and their products such as the skins of red grapes, peanuts and blueberries.^19^, ^20^ The varied bioactivity of this phytochemical has been shown to promote health in a number of ways, including acting as an anti‐cancer, anti‐ageing and anti‐inflammatory agent.^21^, ^22^ In addition to a recent report suggesting resveratrol could be used as an inhalant in dried powder form in the treatment of inflammatory lung diseases,^23^ a previous report from our laboratory showed that this compound worked through multiple pathways to protect mice from SEB‐induced ALI.^24^ In this study, we expand upon our previous findings by testing the efficiency of resveratrol to protect mice from a more robust and lethal SEB‐induced ALI in the mouse. In addition, we explored how this natural product modulates microRNA (miR) to initiate changes in SEB‐induced cytokine release and immune cell activation. In particular, miR‐193a (previous ID: miR‐193a‐3p, miRbase.org) was found to be significantly up‐regulated after exposure to SEB, and this miR targets components involved in TGF‐β and apoptotic signalling pathways. Treatment of SEB‐exposed mice with RES showed a marked decrease in expression of this miR. These data suggest that RES may protect SEB‐mediated toxicity by decreasing the expression of miR‐193a, thereby triggering anti‐inflammatory pathways.

## 2 MATERIALS AND METHODS

### 2.1 Experimental animals

C3H/HeJ female mice, aged 6‐8 weeks, were purchased from Jackson laboratories. Before performing any experiment, all mice were housed in specific pathogen‐free conditions for at least 1 week for acclimatization at the AAALAC‐accredited animal facility at the University of South Carolina, School of Medicine (Columbia, SC). All experimental procedures performed were approved by the University of South Carolina Institutional Care and Use Committee (IACUC) according to the guidelines set by the Care and Use of Laboratory Animals of the National Research Council under AUP# 2169.

### 2.2 SEB and resveratrol preparation

SEB was purchased from Toxin Technology INC (FL, USA) and dissolved in sterile phosphates buffer saline (PBS) at a concentration of 2 μg/μL. RES was purchased from Supelco (MO, USA) and stored at 4°C prior to use. RES was prepared immediately before use by suspending in appropriate vehicle, 1% carboxyl methyl cellulose (CMC, Sigma‐Aldrich, USA), as described.^24^, ^25^

### 2.3 SEB‐induced acute lung injury (ALI) and treatment with resveratrol

To study the effects of resveratrol on SEB‐induced ALI, mice were challenged with SEB with a previously described fatal dual‐dose method in C3H/HeJ mice.^25^, ^26^ SEB was intranasally administrated initially at 5 μg (250 μg/Kg BW) diluted in a final volume of 25 μL PBS. During SEB‐administration, anesthetized mice were held vertically as SEB droplets were added directly into the nostril opening, waiting for 1‐3 minutes until all droplets were inhaled. The second dose of SEB was given intraperitoneally in a dose of 2 μg (100 μg/Kg BW) diluted in 100 μL of sterile PBS. RES was given orally at two‐time‐periods at a dose of 100 mg/Kg. The initial resveratrol oral administration through gavage was given 24 hours (Day‐1) prior to SEB exposure, and the second administration was given 90 minutes (Day 0) before the second challenge with SEB. Mice were watched for any distress, and moribund mice were immediately euthanized.

### 2.4 Blood collection and tissue harvesting

Blood was collected from the retro‐orbital vein of isoflurane‐anesthetized animals for serological and haematological investigations. For cytokine analysis, blood was collected at two‐time‐points: 3 and 48 hours post‐second SEB‐dose exposure. Serum was separated from collected blood by centrifugation and stored at −80°C until time of analysis. Tissues (lung and spleen) were harvested at 48 hours post‐SEB exposure. Excised tissues were kept in cold staining buffer (SB), 1x PBS enriched with 2% foetal bovine serum (Atlanta Biological, USA).

### 2.5 Mononuclear cell isolation and T cell subset determination from the lung and spleen

Collected tissues (lung and spleen) were homogenized with a stomacher lab blender (Seward, England) into single‐cell suspensions in cold SB. Red blood cells (RBCs) were removed from samples by suspension in appropriate amount of RBC lysis buffer (Sigma‐Aldrich, USA) for 3‐5 minutes and washed with SB. Samples were then filtered with 100‐μm cell strainers (Fisher Scientific, China). To isolate mononuclear cells from the lungs, we used Ficoll gradient Histopaque‐1077 (Sigma‐Aldrich, USA) as previously described.^16^ Isolated cells from the lungs and spleens were counted with trypan blue stain (Sigma‐Aldrich, USA) using a Neubauer haemato‐cytometer chamber (VWR Scientific, USA). For T cell subset determination, isolated cells were stained with the following antibodies: CD3‐FITC or APC, CD4‐PE/CY7 or APC, and CD8‐PE (Biolegend, USA). Stained samples were analysed with a flow‐cytometer FC500 (Beckman Coulter Inc, USA).

### 2.6 Bronchoalveolar lavage fluid (BALF) collection

BALF was collected as previously described.^16^, ^24^, ^27^ Briefly, euthanized animals were perfused, and the trachea was tied with surgical silk (Ethicon, USA) at the upper region before removing the lungs from the body. One mL of sterile PBS was injected into the trachea (3‐4 times) to flush out BALF from the lungs. Collected BALF was centrifuged to separate the infiltrated cells from the supernatant, which was stored at −80°C until further analysis.

### 2.7 Pulmonary function tests (PFT) and vascular leak measurement

To evaluate the degree of SEB‐induced dysfunction of the respiratory system, Buxco whole‐body plethysmograph (Buxco, Troy, NY, USA) was used to compare respiratory system parameters, such as airway resistance, among the study groups as previously described.^16^ Briefly, each mouse was held in a whole‐body plethysmograph chamber which delivered varying concentrations (6.25, 12.5, 25 and 50 mg/mL) of methacholine (Biomedical, France), an airway constrictor which creates an acute asthma‐like state. Vascular leak and tissue permeability were measured as previously described.^17^ Evans blue dye (Sigma‐Aldrich, USA) (1%) was injected (100 μL) intravenously 2 hours prior to sacrificing experimental mice, and then the lung lobes were excised and stored in formamide (Fisher Scientific, USA) for 48 hours at 37°C.

### 2.8 Histopathological evaluation

Harvested lungs were fixed in 4% paraformaldehyde (Sigma‐Aldrich, USA) overnight before being embedded into paraffin and obtaining 5‐μm sections for haematoxylin and eosin (H&E) staining. H&E slides were imaged and analysed with Cytation‐5 microscope (Biotek, USA) and Metamorph Software (Molecular Devices, USA).

### 2.9 Detection of cytokines/chemokines by enzyme‐linked Immunosorbent Assay (ELISA)

All cytokines and chemokines (IL‐2, IL‐4, IL‐6, MCP‐1, IFN‐γ, TNF‐α and free active TGF‐β) were detected with commercial mouse‐specific ELISA kits purchased from Biolegend, and the manufacturer's instructions were followed. Detection of cytokines/chemokines, which was measured by absorbance at 450 nm wavelength, was performed using a Victor 2 (Perkin Elmer Life Sciences, USA) plate reader. For detection of cytokines/chemokines in the sera, blood was collected from experimental mice at 3 and 48 hours post‐SEB exposure. For detection of cytokines in the BALF, these samples were collected 48 hours after exposure to SEB.

### 2.10 microRNA (miR) array and in silico analysis

Total RNA isolation was performed with miRNeasy Mini Kit (Qiagen, USA) according to the manufacturer's protocol. RNA concentration and qualification were calculated by Nanodrop spectrophotometer (ThermoFisher Scientific, USA) before analysis by GeneChip Array (miRNA‐4_0, Affymetrix). The Affymetrix GeneChip miRNA 4.0 platform contains 3163 mouse pre‐miRNA and miRNA probes and uses a FlashTag biotin HSR hybridization technique which was performed as previously described.^28^ Data from the microarray were then analysed further with in silico analysis software, Ingenuity Pathway Analysis (IPA, http://www.ingenuity.com/) to determine what gene targets and signalling pathways were being affected by dysregulated miRNA following SEB exposure and subsequent treatment. Predicted miRNA‐target gene alignments were determined with online miRNA database (http://www.microrna.org).

### 2.11 miR validation and target gene quantification by PCR

To validate the miR observations from the microarray and quantitate genes of interest, complementary DNA (cDNA) was prepared from isolated RNA with miScript II RT kit (Qiagen, USA), and qRT‐PCR (Bio‐Rad, USA) was used to determine expression levels. To validate miR, mouse‐specific miRNA primers were purchased from Qiagen, and PCR reactions were performed using miScript II primer assay (Qiagen, USA). The following primer was used: mmu‐miR‐193a‐3p (5′‐AACUGGCCUACAAAGUCCCAGU‐3′). To quantitate gene expression, custom primers were designed and purchased from Integrated DNA Technologies (IDT), and SSO Advanced SYBR^®^Green (Bio‐Rad, USA) was used for the subsequent PCR reactions. The following custom primers were used: TGF‐β1 (forward: 5′‐CTTCAATACGTCAGACATTCGGG‐3′, reverse: 5′‐ GTAACGCCAGGAATTGTTGCTA‐3′), TGFB2 (forward: 5′‐TCGACATGGATCAGTTTATGCG‐3′, reverse: 5′‐ CCCTGGTACTGTTGTAGATGGA‐3′), TGFBR3 (forward: 5′‐GGTGTGAACTGTCACCGATCA‐3′, reverse: 5′‐ GTTTAGGATGTGAACCTCCCTTG‐3′) and DR6 (forward: 5′‐GCCATGTTGACCGTACCACT‐3′, reverse: 5′‐ CAGACTCGCAGGCTCATGTT‐3′).

### 2.12 Transfection experiments with miR‐193a mimic and inhibitor

Transfection of SEB‐activated splenocytes cultures with miR‐193a mimic and inhibitor was performed as previously described.^29^ Briefly, splenocytes were cultured in 24‐well plates (2 × 10^5^ cells per well) and activated with 1 μg/mL of SEB for 24 hours. Transfections were performed with Qiagen HiPerfect Transfection Reagent and 20 nmol/L of either syn‐mmu‐miR‐193‐3p‐mimic (5′‐AACUGGCCUACAAAGUCCCAGU‐3′), anti‐mmu‐miR‐193‐3p‐inhibitor (5′‐AACUGGCCUACAAAGUCCCAGU‐3′) or transfection reagent alone (mock). Transfection efficiency was validated with qRT‐PCR for miR‐193a, and expression of DR6, TGFβ2 and TGFβR3 after transfection and in infiltrated cells was also determined with this method.

### 2.13 Western blotting

Western blot was performed according to the Abcam protocol. Briefly, transfected splenocytes with mimic or inhibitor of miR‐193a or mock only were washed twice with ice‐cold PBS then resuspended in radioimmunoprecipitation assay (RIPA) buffer and maintained with constant agitation for 30 minutes at 4°C to collect protein from the cell lysate. Protein concentration was measured by a Qubit 3.0 fluorometer, (Life Technologies) then loaded into 10% Mini protean precast gels (Bio‐Rad) at a concentration of 20 μg per well followed by electrophoresis for 90 minutes at 120 volts. Protein bands were transferred from the gel to iBlot 2 NC Mini Stacks membrane (Invitrogen) by iBlot gel transfer device (Invitrogen) set on 20 volts for 7 minutes. Membranes were incubated overnight at 4°C with primary antibody rat anti‐mouse TGFβ2 (R&D systems), goat anti‐mouse TGFβR3 (R&D systems) or rabbit anti‐mouse TNFRSF21/DR6 (LifeSpan Bioscinces). The housekeeping gene was goat anti‐GAPDH (Biolegend). Images of protein bands were taken with a C‐Digit scanner (LI‐COR) after staining of blotting membrane with WesternSure Chemiluminescent substrate (LI‐COR). Quantification of target proteins was calculated by ImageJ software, background was subtracted, protein quantity was normalized to the housekeeping gene, and the target proteins density was calculated relative to expression of respective protein in mock group.

### 2.14 Statistical analysis

Graphpad prism 6.0 software was used for statistical analysis, and built‐in one‐way ANOVA (Tukey's multiple comparisons tests) and multiple *t* test comparisons with Holm‐Sidak correction method were applied. Each experimental group was composed of 4‐5 mice. *P* < .05 was considered to be statistically significant.

## 3 RESULTS

### 3.1 Resveratrol attenuates clinical parameters after exposure to “dual dose” of SEB

**Figure 1:**
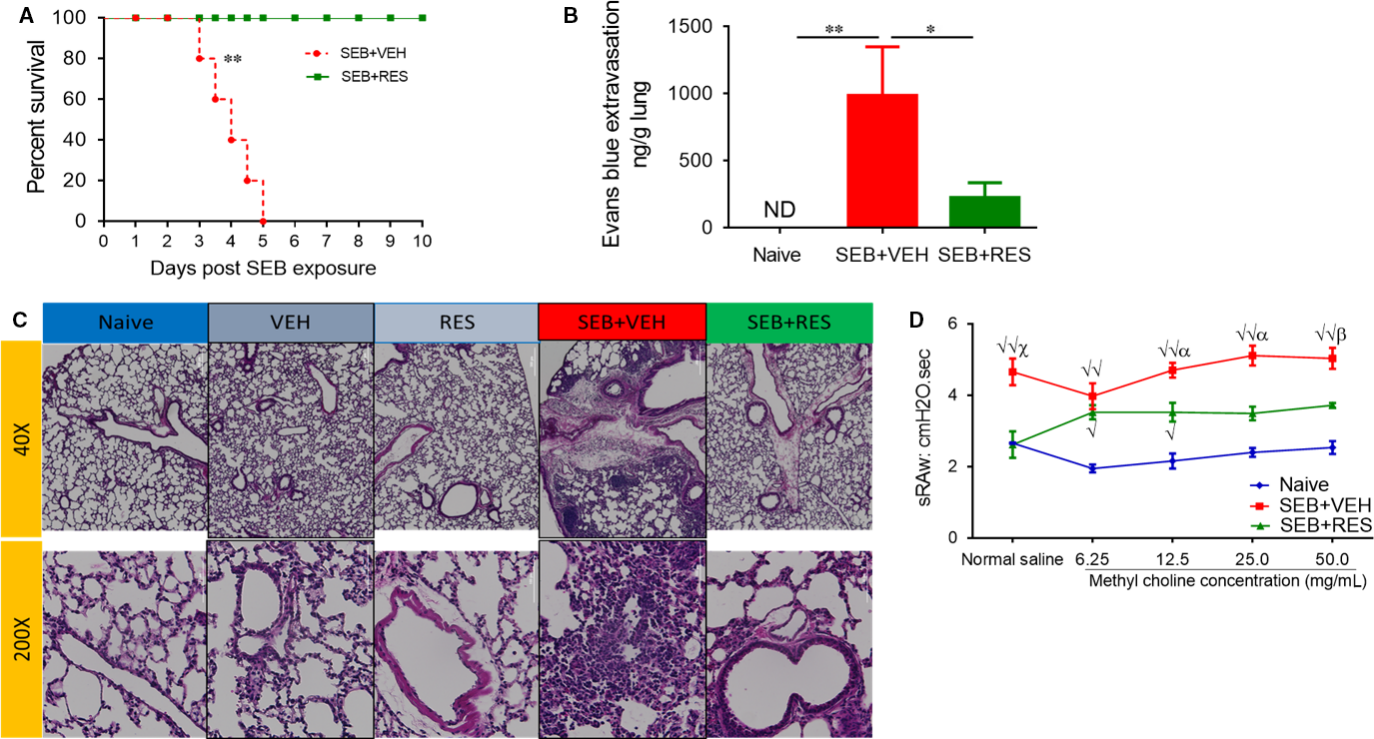
Effect of RES on clinical parameters of SEB‐induced ALI. Mice were given two doses of SEB as described in Methods. The first dose of RES was given 24 h prior to SEB exposure (day‐1) and the second at 90 min (Day 0) before the second challenge with SEB. The mice were studied for the next 10 d. (A) Survival curve over 10 d for SEB + VEH (n = 5) and SEB + RES (n = 5) groups. (B) Concentration of Evans blue dye in isolated lungs was used to study vascular permeability. (C) Representative histopathological H&E slides of excised lungs; (D) Pulmonary function tests depicting specific airway resistance (sRaw). Significance was measured in panel D only as following: √ = *P* < .01, √√ = *P* < .0001 in comparison between Naïve and SEB + VEH or SEB + RES groups. α = *P* < .05, β = *P* < .01, χ = *P* < .001 in comparison between SEB + VEH and SEB + RES groups in panel D. Significance in panels A and B was depicted as follows: *P < .05, **P < .01

**Figure 2:**
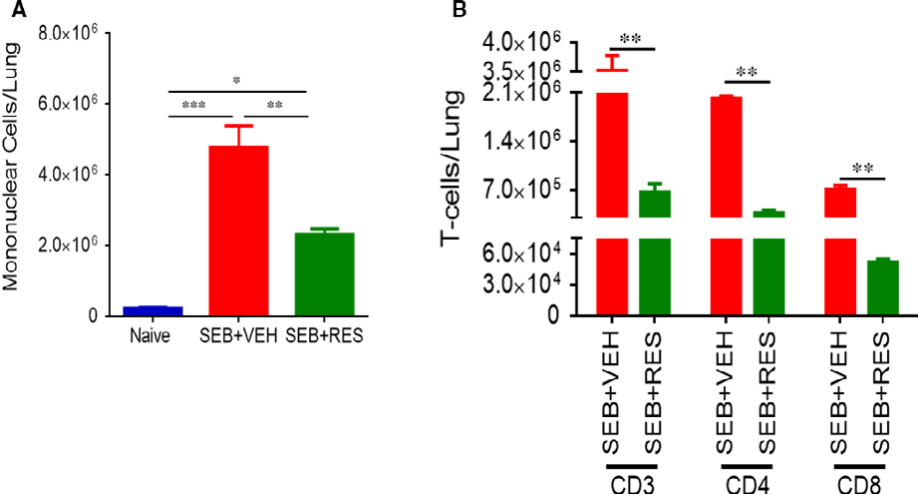
Resveratrol (RES) decreases cellular infiltration in the lungs of SEB‐treated mice. Mice were exposed to SEB and treated with RES as described in Figure 1 legend. (A) Total number of mononuclear cells isolated from the lungs. (B) Total number of T cell subsets analysed after antibody staining and flow cytometry analysis. Significance was depicted as follows: *P < .05, **P < .01, ***P < .001

We first determined whether RES would protect mice in the dual‐dose model of SEB exposure, which leads to almost 100% mortality as shown by us previously.^27^ As shown in Figure 1A, 100% of mice exposed to SEB + VEH died within the 5‐day period. Also, such mice started showing signs of weakness by 24 hours. Interestingly, 100% of the mice that were given treatment with RES survived for more than 10 days after SEB exposure, and there were no visible clinical signs in mice that survived. To study vascular leak in the lungs following SEB exposure, we injected Evans blue dye intravenously (i.v.) as described^17^ and found that lungs from the SEB + VEH group showed increased extravasation of the dye, indicative of vascular leak while treatment with RES significantly reduced the vascular leak (Figure 1B). Histopathological evaluation of the lungs after 48 hours post‐SEB + Vehicle exposure showed that the lung architecture was clearly altered compared to naïve lungs, with excessive cellular infiltration in the alveolar and interstitial spaces of the tissue (Figure 1C). In contrast, mice given RES showed a marked decrease in this SEB‐driven cellular infiltration into the lungs, closely resembling lungs from naïve mice. Accumulation of inflammatory cells in the pulmonary gas‐exchange area greatly affects the respiratory system performance, often leading to an increase in resistance of airway tracts, extending the required time to move air from the chest cavity outward and verse versa. To determine this airway resistance, we performed whole‐body plethysmography in SEB‐exposed mice. Measurements showed mice given SEB had increased specific airway resistance (sRaw; Figure 1D) values when compared to naïve mice, which is indicative of impaired pulmonary function. In contrast, treatment with RES caused significant decrease in these values. Collectively, these data demonstrated that RES was able to significantly ameliorate key clinical parameters of lethal SEB‐mediated lung injury. SEB + VEH‐treated mice showed significant increase in the total number mononuclear cells in the lungs when compared to naïve mice. In contrast, SEB + RES group demonstrated a significant decrease in the number of mononuclear cells (Figure 2A). Also, RES treatment decreased the accumulation of T cells in the lung after exposure to SEB. When T cell subsets were analysed in the lungs, the SEB + RES group demonstrated a significant decrease in CD3^+^, CD4^+^ and CD8^+^ T cells when compared to SEB+ Vehicle group (Figure 2B). Our previous studies demonstrated that resveratrol alone does not cause any changes in the immune response in naïve animals.^24^ It is for this reason, we did not include this group. However, we did include this vehicle or resveratrol alone for histopathological studies of the lungs, which showed no significant changes when compared to naïve animals (Figure 1C).

### 3.2 Resveratrol alters cytokine levels brought on by “cytokine storm” after exposure to SEB

**Figure 3:**
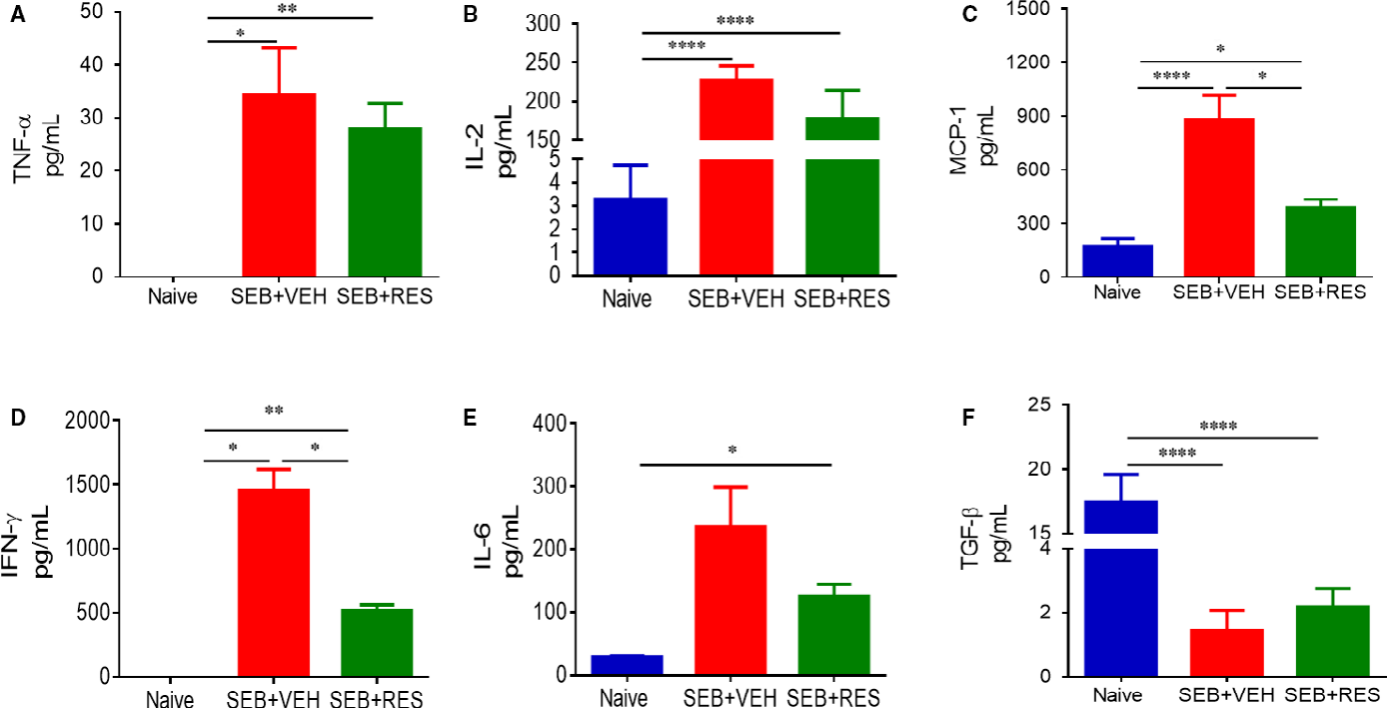
Effect of RES on serum cytokines induced by SEB. Mice were treated with SEB and RES as described in Figure 1 legend. Serum was collected from all experimental groups (Naïve, SEB + VEH, SEB + RES) 3 h after second dose of SEB. ELISA kits were used to detect (A) TNFα, (B) IL‐2, (C) MCP‐1, (D) IFN‐γ, (E) IL‐6 and (F) TGF‐β. Significance was depicted as follows: *P < .05, **P < .01, ***P < .001, ****P < .0001

**Figure 4:**
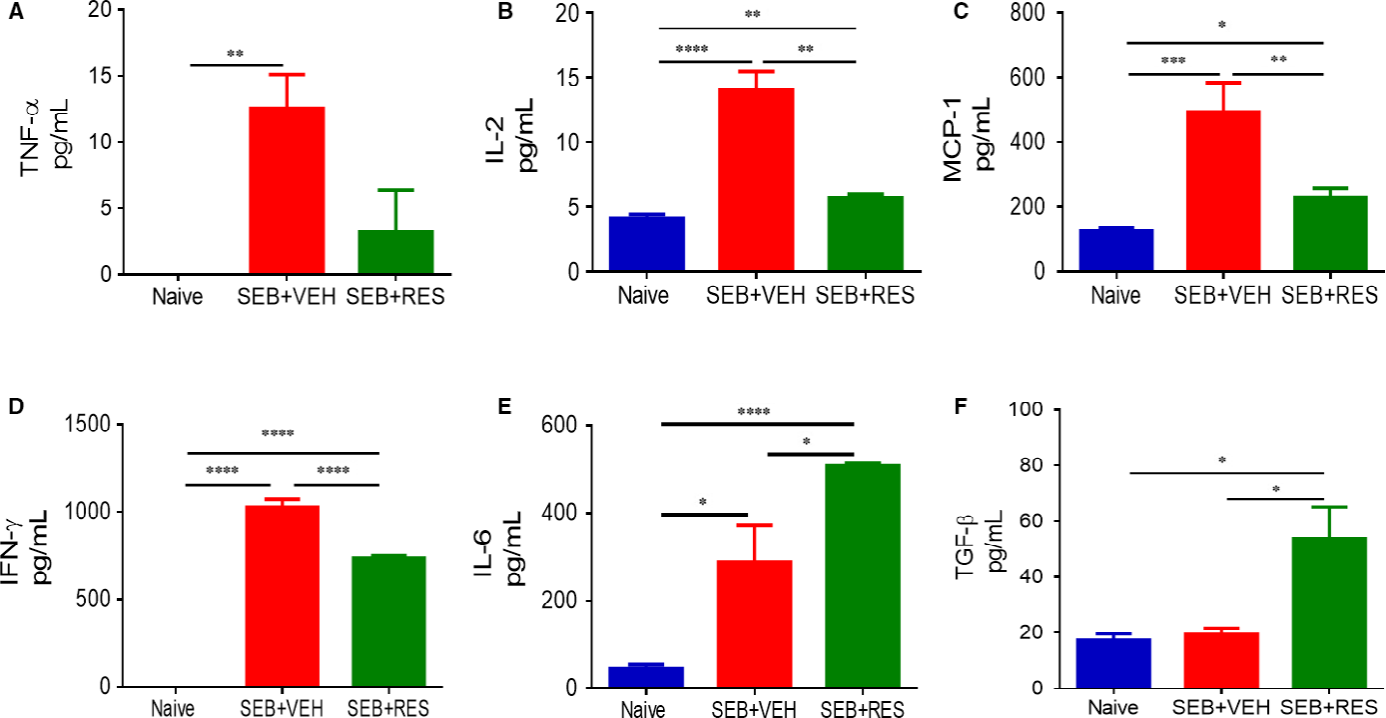
Effect of RES on SEB‐induced serum cytokine levels at 48 h. Serum was collected from all experimental groups (Naïve, SEB + VEH, SEB + RES) 48 h after second dose of SEB. ELISA kits were used to detect (A) TNFα, (B) IL‐2, (C) MCP‐1, (D) IFN‐γ, (E) IL‐6 and (F) TGF‐β. Significance was depicted as follows: *P < .05, **P < .01, ***P < .001, ****P < .0001

To investigate the effect of RES on the cytokine storm induced by SEB, we studied the levels of cytokines in the serum at 3 (Figure 3A‐F) and 48 (Figure 4A‐F) hours after the second exposure to SEB. At 3 hours, post‐SEB exposure, TNF‐α, IL‐2, MCP‐1, IFN‐γ and IL‐6 were all significantly up‐regulated in the SEB + VEH group when compared to naïve mice, and furthermore, treatment with RES caused a marked decrease in all of these cytokine levels. In contrast, at 3 hours, we detected very low levels of anti‐inflammatory, TGF‐β, in all groups. By 48 hours, in SEB + VEH group, some of the inflammatory cytokines studied had dropped significantly (TNF‐α, IL‐2), while levels of others (MCP‐1, IFN‐γ and IL‐6) still remained high (Figure 4). Moreover, RES treatment led to decrease in all inflammatory cytokines at 48 hours except IL‐6 (Figure 4). Interestingly, we found that while TGF‐β levels were low in SEB‐treated groups and similar to the naïve mice, treatment with RES caused a significant increase in TGF‐β (Figure 4F).

**Figure 5:**
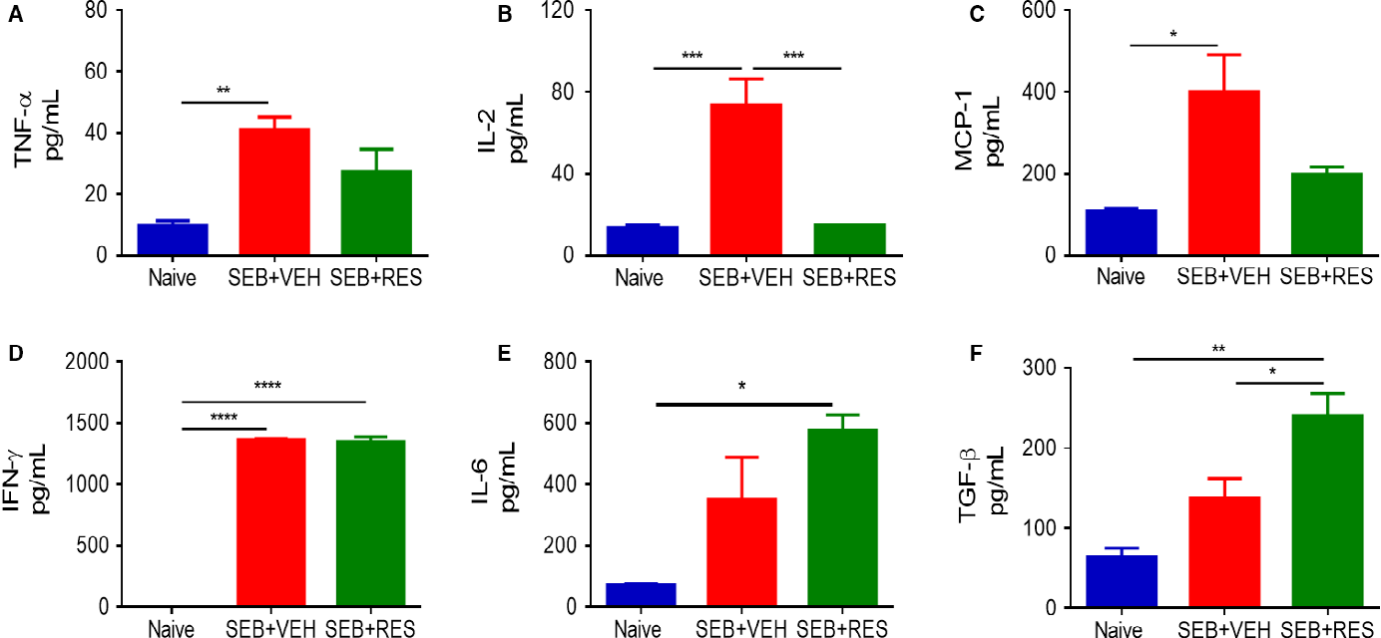
Effect of RES on SEB‐induced BALF cytokine levels at 48 h. BALF was collected from all experimental groups (Naïve, SEB + VEH, SEB + RES) 48 h after second dose of SEB. ELISA kits were used to detect (A) TNFα, (B) IL‐2, (C) MCP‐1, (D) IFN‐γ, (E) IL‐6 and (F) TGF‐β. Significance was depicted as follows: *P < .05, **P < .01, ***P < .001, ****P < .0001

In the BALF, we observed a similar increase in inflammatory cytokines such as TNF‐α, IL‐2, MCP‐1 and IFN‐γ in the SEB + VEH group when compared to naive mice, and treatment with RES was able to significantly reduce all inflammatory cytokines except IFN‐γ and IL‐6 (Figure 5). Interestingly, in the BALF, we noted a marked increase in TGF‐β levels in SEB + VEH mice when compared to naïve mice, and SEB + RES groups showed an additional increase in this cytokine.

### 3.3 Resveratrol alters miR profile in lung‐associated immune cells after exposure to SEB

**Figure 6:**
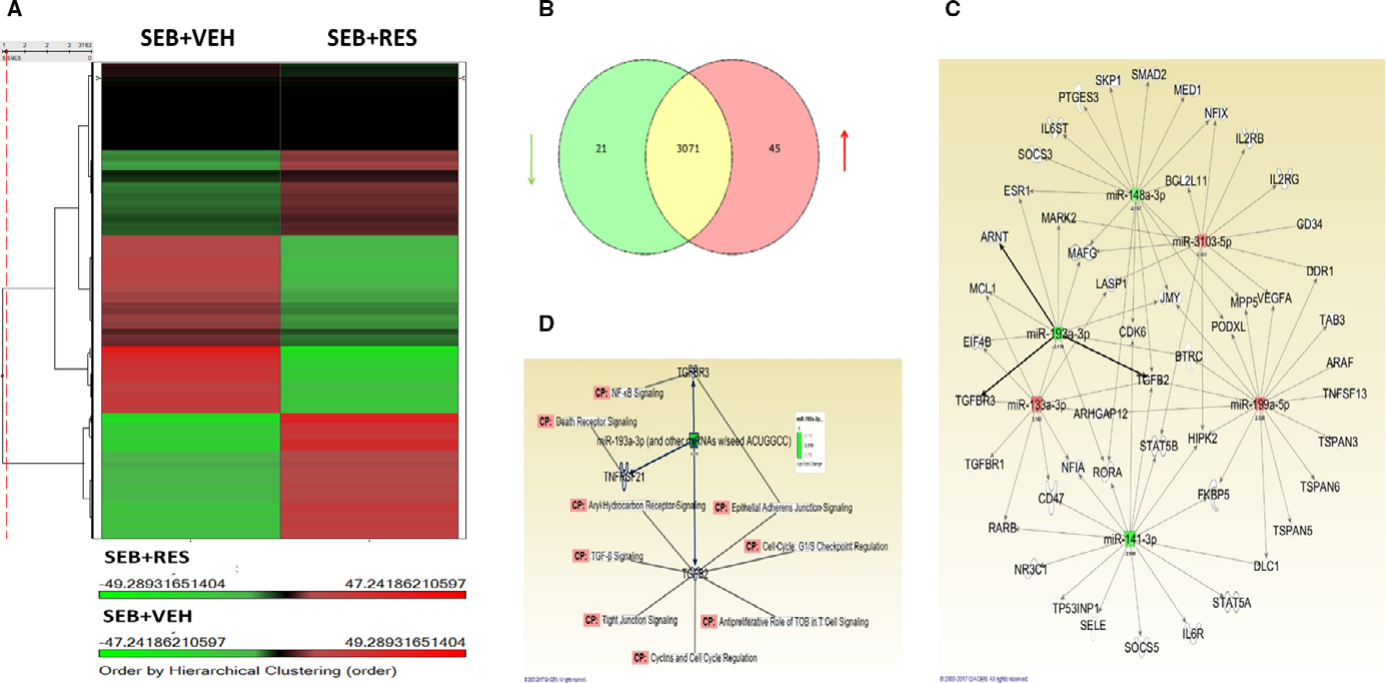
Resveratrol (RES) alters miRNA expression profiles in mononuclear cells isolated from SEB‐exposed mice. Mononuclear cells were isolated from the lungs of mice treated with SEB + VEH or SEB + RES as described in Figure 1 legend, and miRNA array was performed using GeneChip miRNA 4.0 platform. (A) Heatmap comparing SEB + VEH and SEB + RES groups with miRNA expression profiles (green indicates less intensity and red indicates an increase). (B) Venn diagram depicting fold changes of 3137 detected miRs that were either up (red), down (green) or not changed (yellow) significantly when comparing SEB + VEH and SEB + RES groups. Significant changes were determined to be greater or lesser than 2 linear fold change. (C) IPA‐derived network depicting the relationship of altered miR to their potential gene targets. (D) IPA‐derived network depicting the relationship of miR‐193a to altered signalling and regulatory pathways

To investigate the role of microRNA, we used miR array on mononuclear cells isolated from the lungs and compared the profiles in the SEB + VEH vs SEB + RES group. Figure 6A shows a heat map depicting differential expression of detectable miRs performed with the Genechip miRNA 4.0 array platform (http://www.affymetrix.com). Of the 3137 detectable miRNAs within the samples, 66 microRNAs were found to be significantly dysregulated (greater than 2 linear fold change) in the SEB + RES treatment group when compared to the SEB + VEH group, with 21 miRs being down‐regulated and 45 miRs up‐regulated (Figure 6B). Next, the IDs and fold change quantities of the 66 differentially regulated miRNA were analysed with IPA on line software from Qiagen (http://www.ingenuity.com/) to determine potentially affected gene targets (Figure 6C) and molecular pathways (Figure 6D). In Figure 6C, a representative network generated by IPA analysis shows how several dysregulated miRs (miR‐133, 141, 148a, 193a, 199a and 3103) target key regulators of cellular processes and immunity. Interestingly, miR‐193a was the most down‐regulated (‐3.178‐fold change), and this miR targeted genes linked to TGF‐β (TGFβR1, TGFβR3 and TGFβ2), which we found to be significantly up‐regulated in BALF (Figure 5F) following RES treatment. Focusing on miR‐193a, we found through IPA analysis that this miR affected many pathways involved in immune regulation including death receptor signalling, aryl hydrocarbon receptor (AhR) signalling, cell cycle regulation, NF‐κB signalling pathways and TGF‐β signalling (Figure 6D). In particular, this miR targeted 3 proteins including TGFβ2, TGFβR3 and DR6 (also known as TNFRSF21).

### 3.4 miR‐193a is down‐regulated in RES‐treated mice exposed to SEB, and it targets genes DR6, TGFβ2 and TGFβR3

**Figure 7:**
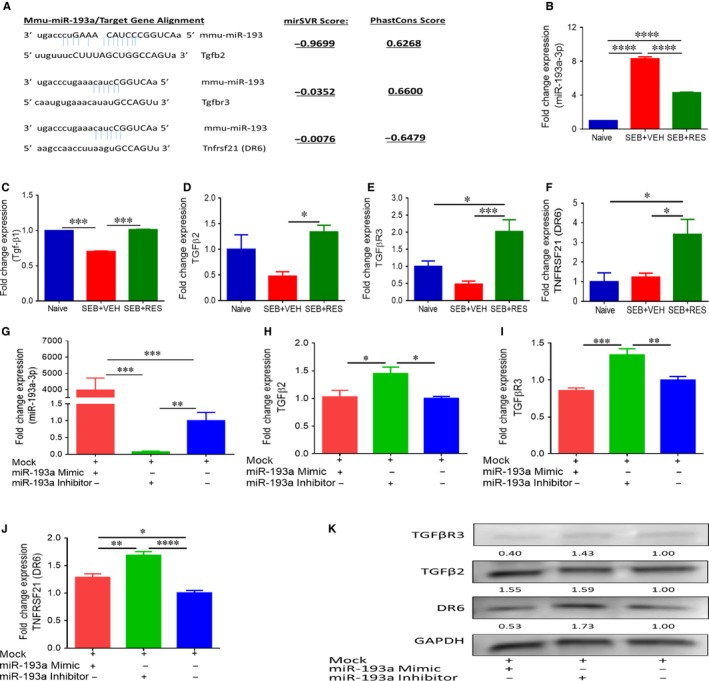
Validation of miR‐193a and its targets. (A) Gene target alignment scores (mirSVR and PhastCons) from microrna.org are depicted for miR‐193a‐3p and gene targets TGFB2, TGFBR3 and DR6. (B) miR‐193a expression was validated with qRT‐PCR from RNA samples collected from lung mononuclear cells from naïve, SEB + VEH and SEB + RES groups. Data are expressed as fold change compared to naïve controls. (C, D, E and F) qRT‐PCR expression levels of TGF‐β1, TGFβ2, TGFβR3 and DR6, respectively, in infiltrated mononuclear cells into lungs of SEB + VEH and SEB + RES groups compared to naïve controls. (G) Validation qRT‐PCR for the expression of miR‐193a‐3p in SEB‐activated splenocytes when transfected with mock, mimic or inhibitor for miR‐193a‐3p. Data are expressed as fold change compared to mock controls. (H, I, and J), qRT‐PCR values from TGFB2, TGFBR3 and DR6 after transfection of SEB‐activated splenocytes with either mock, mimic or inhibitor for miR‐193a‐3p. K, Western blot of TGFβR3 (94 kD), TGFβ2 (52 kD) and DR6 (23.7 kD) proteins extracted from splenocytes transfected with mimic or inhibitor of miR‐193a or mock. Numbers below blots indicate the densitometry of the bands when normalized to GAPDH protein. Significance was depicted as follows: *P < .05, **P < .01, ***P < .001, ****P < .0001

Next, we studied the miR‐target alignment miRSVR and PhastCons scores (microrna.org) for miR‐193a and the potential target proteins, TGFβ2, TGFβR3 and DR6. The scores shown in Figure 7A indicated that miR‐193a had a high probability of targeting these proteins, although the scores for DR6 were somewhat weaker compared to TFGβ2 and TGFβR3 (Figure 7A). Next, we validated the expression of miR‐193a with qRT‐PCR. As shown in Figure 7B, miR‐193a expression was significantly higher in cells from SEB + VEH group when compared to naïve controls, and it was reduced by half in the SEB + RES group, confirming the observations from the array results that RES was down‐regulating miR‐193a expression. As many of these targets were linked to TGFβ and we observed alterations in TGF‐β in the SEB + RES‐treated group, we determined expression of this cytokine in the mononuclear cells isolated from the lungs. The data showed that cells from the SEB + RES group had significantly higher expression of TGFβ1, TGFβ2, TGFβR3 and DR6 when compared to SEB + VEH group (Figure 7C‐F, respectively). Next, we performed transfection experiments to validate the miR‐target interactions. Data shown in Figure 7G indicated that SEB‐activated splenocytes transfected with mock, mimic or inhibitors for miR‐193a showed the expected results in terms of the expression of miR‐193a. Furthermore, transfecting these SEB‐activated cells with miR‐193a‐3p resulted in direct dysregulation of the proteins of interest. Specifically, increasing the levels of this miR with the mimic led to significant decreases in the expression of TGFβ2, TGFβR3 and DR6 when compared to those cells that were transfected with the inhibitor, thereby validating the in silico data obtained (Figure 7H‐J, respectively). Protein expression of TGFβ2, TGFβR3 and DR6 was higher in cells that were transfected with inhibitor when compared to transfected cells with mimic and normalized to mock (Figure 7K). Taken together, these experiments showed that miR‐193a‐3p levels are altered by RES under SEB‐activated conditions, and this, in turn, could lead to an up‐regulation of normally targeted proteins TGFβ2, TGFβR3 and DR6, which are involved in important processes, such as TGF‐β and NF‐κB signalling.

## 4 DISCUSSION

SEB, produced by Gram‐positive bacteria *S. aureus*, is a superantigen because of its ability to activate a large proportion of T cells.^10^ Such activation leads to cytokine storm and consequently considerable damage to internal tissues.^13^ Thus, SEB poses a significant threat, which is why the CDC has classified it as category B select agent with the potential of being used as a biological weapon.^11^, ^12^ Exposure to SEB in humans can lead to food poisoning as well as fatal toxic shock. Inhalation of SEB can cause significant damage to the lungs resulting in respiratory failure.^16^, ^17^ In the current study, performed with the 100% lethal dual‐exposure model to SEB, we demonstrate that treatment with RES can completely rescue the mice from mortality. Our data suggest that this may result from the potent suppression of inflammation mediated by RES.

Resveratrol has been used as a therapeutic agent for thousands of years and has been a staple in Eastern medicine, known as ko‐jo‐kon, to treat aliments of the heart, liver, blood vessels and various other organs.^30^ This compound is a well‐known ligand for the AhR, and AhR activation has been shown to promote anti‐inflammatory Treg differentiation while suppressing pro‐inflammatory Th17 cells.^31^ In the current study, we demonstrate that RES can act as a potent anti‐inflammatory agent in combating the negative effects such as cytokine storm associated with inhalation of SEB, which can result in a fatal ALI condition. This study reinforces our previous results highlighting RES use in a much more acute and severe model of SEB‐induced ALI.^24^ Our data demonstrate that RES was able to decrease several SEB‐mediated inflammatory cytokines in the lungs and blood, thereby preventing lung injury.

Resveratrol has been shown to be effective against a variety of different types of inflammatory lung disorders.^32^, ^33^, ^34^, ^35^, ^36^ In many of these studies, RES was found to dampen the production of inflammatory cytokines and modulate a number of factors involved in inflammatory signalling pathways, such as toll‐like receptor 4 (TLR4), myeloid differentiation primary response gene 88 (myd88) and nuclear factor‐kappa B (NF‐κB).^37^ Interestingly, a recent study showed that a RES‐curcumin hybrid mix was able to decrease production levels of TNF‐α, IL‐6 and IL‐12 in the lung caused by LPS.^32^ Despite clear indication that RES is a potent immunosuppressant in lung inflammatory and injury models, the precise mechanisms of action of RES remain unclear. It is for that reason that we investigated the role of miRNA in the lung injury model.

In the present study, we found that RES treatment altered the expression of miR signatures of immune cells under SEB‐activated conditions. Previous studies have shown that RES can regulate the expression of miRs and such a property has been linked to the ability of this natural compound to regulate inflammation and inflammatory diseases.^38^, ^39^, ^40^, ^41^ For example, miR‐101b and miR‐455, thought to target inflammatory processes, were found to be up‐regulated in colitis‐associated tumour model after treatment with RES.^42^ miR‐217, increased in response to high glucose in rat glomerular mesangial cells, was reduced following treatment with RES, thereby leading to a restrained inflammatory response.^43^ SEB itself was shown by our laboratory to significantly alter miRs to promote ALI through induction of miR‐155 to suppress SOCS1, a negative regulatory of cytokine signalling.^44^

In the current study, we found miR‐193a to be up‐regulated upon exposure to SEB but was significantly down‐regulated after treatment with RES. Interestingly, current literature shows miR‐193a to be involved in both anti‐inflammatory and pro‐inflammatory properties. For example, miR‐193a was reduced in intestinal inflammation in colitis;^45^ however, our own laboratory showed that this miR may target inflammatory IL‐12 in a study on patients with post‐traumatic stress disorder (PTSD).^46^ This is possible because any given miR may regulate many processes by targeting different molecules. In that context, it is interesting to note that in the current study, we identified that miR‐193a may target TGF‐β pathway.

In the current study, we noted that at 3 hours post‐SEB exposure, while inflammatory cytokines in the blood were highly induced, TGF‐β levels were very low. However, at 48 hours, the TGF‐β levels increased compared to 3 hours, while RES caused an additional increase in its levels. We also noted that RES caused marked increase in TGF‐β levels in the BALF as well. TGF‐β is known to act as a potent anti‐inflammatory agent, and one of the mechanisms of immunosuppression could include its ability to induce Tregs.^47^ In addition, we found that miR‐193a targets components of the TGF‐β signalling pathway, mainly TGFβ2 and TGFβR3. Thus, by down‐regulating miR‐193a, RES up‐regulates these targets. TGF‐β has three homologues (TGFβ1, TGFβ2 and TGFβ3) in the mammalian system, and it has been suggested that these variants could have differing effects on signalling pathways. In our study, we showed that miR‐193a directly targeted TGFβ2, which has been shown to attenuate the production of LPS‐induced inflammatory cytokines IL‐6, IL‐1β and TNF‐α by ERK activation.^47^ Interestingly, TGFβR3, one of the three receptor subunits for TGF‐β, has been shown to play an important and necessary role in TGFβ2 signal transduction.^48^ In this regard, RES through down‐regulation of miR‐193a could be playing dual roles in anti‐inflammatory TGF‐β signalling. Butz et al found that the relationship between TGF‐β and miRNAs biogenesis is an autoregulatory feedback loop, as many miRNAs may target a single gene, a single miRNA can target multiple genes.^49^ Also, in a study of lung cancer, the researcher found there is a negative regulatory model between the expression of miR‐193 and TGF‐β‐induced lung cancer.^50^ Down‐regulation of this miR could lead to increased production of TGFβ2, and its signal transduction could be further enhanced by the presence of increased TGFβR3. This combination may, in turn, lead to decreased cytokine production induced by SEB.

Another identified and validated target for miR‐193a in our studies was DR6 (also known as TNFRSF21), which is involved in apoptosis and activation of NF‐κB.^51^, ^52^ In CD4^+^ T cells, knock‐down of DR6 leads to hyper proliferation in response to both TCR‐mediated and protein‐antigen stimulation.^53^ Therefore, to control or negatively regulate T cell proliferation, DR6 should be present. In connection to our findings, miR‐193a is up‐regulated in the presence of SEB; thus, targeted DR6 would be down‐regulated. This could help explain how T cells are able to proliferate and survive after being exposed to SEB. However, upon treatment with RES, this effect is reversed, leading to decreased miR‐193a and increased DR6, thereby halting T cell proliferation. These data are consistent with our observation that RES treatment in SEB‐exposed mice caused significant a decrease in T cell numbers in the lungs.

Taken together, the current study demonstrates that RES can successfully attenuate SEB‐mediated inflammatory processes, acute lung injury and mortality in mice, thereby demonstrating that it is a highly potent anti‐inflammatory agent. This is especially important as SEB‐inhalation by humans can produce lethal toxic shock, yet there is little to no treatment options other than standard supportive care. We also identify a potential and novel mechanism to explore, and that is the modulation of miRs, specifically miR‐193a, which has not received much attention yet as a molecule involved in blocking inflammation when compared to other more well‐known miRs. As highlighted in this work, this miR could be involved in the regulation of TGF‐β pathways, a key modulator of inflammation. miR‐193a‐3p could also be a very promising potential therapeutic target not only in SEB‐mediated diseases but also in other inflammatory diseases where TGF‐β is often implicated.

## CONFLICT OF INTERESTS

The authors confirm that there are no conflict of interests.
